# Green Mild Acid Treatment of Recycled Concrete Aggregates: Concentration Thresholds for Mortar Removal While Avoiding Degradation of Original Limestone Aggregate and Concrete

**DOI:** 10.3390/ma18153673

**Published:** 2025-08-05

**Authors:** Shunquan Zhang, Yifan Zhang

**Affiliations:** School of Civil Engineering and Architecture, Engineering Research Center of Anhui Metallurgical Solid Waste Green Construction, Anhui University of Technology, Ma’anshan 243032, China

**Keywords:** recycled concrete aggregate, original natural coarse aggregate, acetic acid immersion, microstructure, interfacial transition zone

## Abstract

While acetic acid has proven effective as a mild acidic treatment for removing adhered mortar from recycled concrete aggregate (RCA) surfaces, its potential for dissolving damage to the surface of the original natural coarse aggregate (NCA) within the RCA and its impact on the resultant concrete properties require careful consideration. This investigation systematically evaluates the effects of varying concentrations of dilute acetic acid solutions, commonly used in RCA treatment protocols, through a multi-methodological approach that includes comprehensive physical characterization, stylus and 3D optical profilometry, scanning electron microscopy (SEM), and nanoindentation analysis. The results show that even dilute acid solutions have an upper concentration limit, as excessive acid concentration, specifically 0.4 M, induces significant textural dislocations on NCA surfaces, creating millimeter-scale erosion pits that increase aggregate water absorption by 18.5%. These morphological changes significantly impair concrete workability and reduce compressive strength performance. Furthermore, microstructural analysis reveals a 45.24% expansion in interfacial transition zone (ITZ) thickness, accompanied by notable reductions in elastic modulus and microhardness characteristics. In practical RCA treatment applications, for RCA containing limestone-based NCA, it is recommended to use acetic acid concentrations between 0.1 and 0.3 M to avoid substantial physical and microstructural degradation of aggregates and concrete.

## 1. Introduction

Recycling waste concrete into recycled aggregates offers significant environmental benefits. It addresses the ecological deterioration caused by massive concrete waste discharge [1,2,3]. Simultaneously, it mitigates severe scarcity of natural sand and gravel resources [4,5,6,7]. However, the processing of recycled aggregates remains economically and technically challenging in practice. The loose and porous old mortar adhered to the surface of recycled concrete aggregate (RCA) causes the RCA to have higher porosity, water absorption, and crushing index than natural coarse aggregate (NCA), limiting the use of RCA [2,8,9]. Acid immersion effectively eliminates old mortar from the surface of RCA, improving its quality [10,11].

To enhance the quality of RCA, viable approaches focus on removing adhered mortar through mechanical grinding, thermal treatment via high temperature, and chemical dissolution by acid immersion, collectively reducing mortar content to improve RCA performance [2,9]. The acid immersion method requires no special mechanical equipment and consumes minimal energy, with a specific concentration of the acid solution often employed to dissolve old mortar [12]. However, the sulfuric and hydrochloric acids typically used in acid immersion are hazardous to humans, and the resulting waste solution contaminates the environment [13,14]. Additionally, residual Cl^−^ and SO_4_^2−^ on the surface of the immersion aggregate undermine the durability of the concrete structure [10,15,16,17,18]. Dilute acetic acid is safer, cleaner, and less expensive than other acids, for example, vinegar, making it suitable for treating RCA in a gentle, environmentally friendly manner [19]. The dilute acetic acid treatment of RCA leaves no residual ions on the surface that could harm the environment or the concrete. Compared to more potent acids, the treated RCA requires no water washing, conserving water resources and reducing treatment costs. Furthermore, the waste solution from the treatment of RCA with dilute acetic acid can be recycled as mixing water for cement and concrete [12,19] and can also be transformed into high-value-added vaterite (a special crystal primarily made of CaCO_3_) through CO_2_ sequestration, which may be used to produce regenerated acetic acid [20,21].

The dilute acetic acid immersion technique depends on two factors: immersion duration and acid concentration. Existing research has shown that the mortar removal impact of RCA may be maximized by immersing it in dilute acetic acid for 24 h, which is a short and efficient immersion duration that meets the needs of actual projects [12,21]. The concentration of acid, a crucial factor in the acid treatment procedure, significantly influences the quality improvement of RCA. The H^+^ in the acid solution combines with the hydration products in the old mortar on the surface of RCA, forming Ca(OH)_2_, C-S-H gel, and CaCO_3_ [11,12]. The more mortar sticks to the RCA’s surface, the higher the acid concentration required to remove it. If the acid concentration is insufficient, it may fail to dissolve surface pollutants effectively, hence diminishing the treatment’s efficacy. Appropriate acid concentration can improve the surface microstructure of RCA, reduce porosity, water absorption, and crushing index, increase interfacial binding strength with cementitious materials, and improve overall mechanical characteristics [10]. Wang et al. [21] utilized a dilute acetic acid immersion approach to reduce the water absorption of RCA by 9–19%, resulting in treated RCA concrete exhibiting over 25% greater compressive strength compared to untreated RCA. Verma et al. [22] proved that, when 50% RCA treated with dilute acetic acid immersion substituted NCA, the resulting concrete construction met engineering strength and durability criteria. Furthermore, RCA treated with dilute acetic acid immersion may efficiently remove acid-softened and residual mortar from the aggregate surface by low-intensity mechanical vibration, reducing the aggregates’ water absorption [11,23,24].

However, a critical trade-off exists when optimizing acid concentration solely for mortar removal efficacy. Limestone natural aggregate is extensively utilized as coarse aggregate for concrete in the construction industry, owing to its widespread availability, ease of mining and processing, and outstanding performance as a concrete aggregate [25,26,27]. While it is recognized that original limestone NCA, formed through complex and lengthy geological processes, is generally less reactive to acid than synthetic old mortar [10], this inherent difference in reactivity has led to an oversight. The potential detrimental effects of elevated acetic acid concentrations on the integrity of the original limestone NCA embedded within the RCA have been largely neglected.

When the acid concentration becomes too high, it can dissolve not only the old mortar but also damage the main aggregate. The old mortar does not usually cover the surface of the RCA completely, which means both the original NCA and the old mortar are exposed to the acid solution. Consequently, high acid concentrations, often adopted in practice for faster processing, can inadvertently compromise the quality of the original NCA. This degradation of the NCA component subsequently undermines the overall performance of the concrete produced with the treated RCA. Critically, there is a lack of quantitative understanding regarding the degradation thresholds of common limestone NCA under practical dilute acetic acid treatment conditions.

This study employed widely used limestone NCA to simulate the original aggregate within RCA, systematically immersing it in dilute acetic acid solutions (0.1, 0.2, 0.3, and 0.4 M) for 24 h to quantitatively evaluate acid concentration effects on the NCA’s properties and resultant concrete. Therefore, the contribution of this work lies in establishing degradation thresholds for limestone-based NCA under acetic acid treatment. This provides essential scientific guidelines to reconcile effective mortar removal with preservation of original NCA quality, vital for optimizing RCA treatment and ensuring concrete performance.

## 2. Experimental Program

### 2.1. Materials

**Figure 1:**
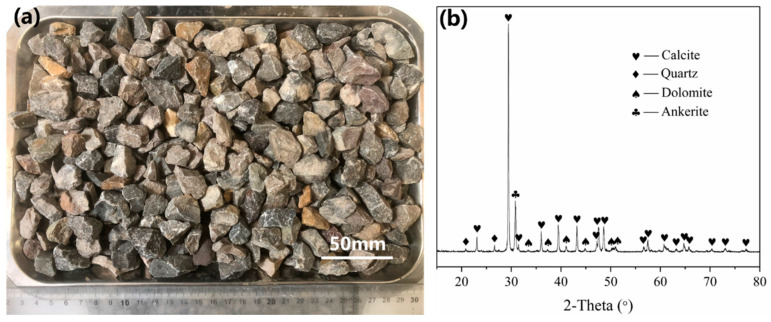
(**a**) Image and (**b**) XRD pattern of original limestone-based NCA.

Natural limestone coarse aggregate with a grain size of 5–20 mm was used as NCA in RCA in this study (Figure 1a). The aggregates were sourced from crushed limestone rocks in Jiangsu Province, China, and had no prior exposure to concrete use. Its apparent density, crushing index, and water absorption are 2.62 g/cm^3^, 7.2%, and 2.7%, respectively. After grinding the natural aggregate into a powder and screening through a 75 μm sieve, the XRD test results showed that the aggregate was a carbonate rock with calcite and dolomite as the principal components (Figure 1b). The cementitious material employed was P·II 52.5 ordinary Portland cement (OPC), with densities of 3150 kg/m^3^ and 365 m^2^/kg. Natural river sand was utilized as the fine aggregate, with particle sizes ranging from 0.08 to 2 mm and a fineness modulus of 2.6. Furthermore, the acetic acid used in this study was dilute acetic acid in solutions of 0.1, 0.2, 0.3, and 0.4 M derived from glacial acetic acid solutions with purity greater than 99.9%, which are commonly used acid concentrations for soaking treatment of RCA and are effective in removing old mortar from the surface of RCA [12,21].

### 2.2. Sample Preparation

**Figure 2:**
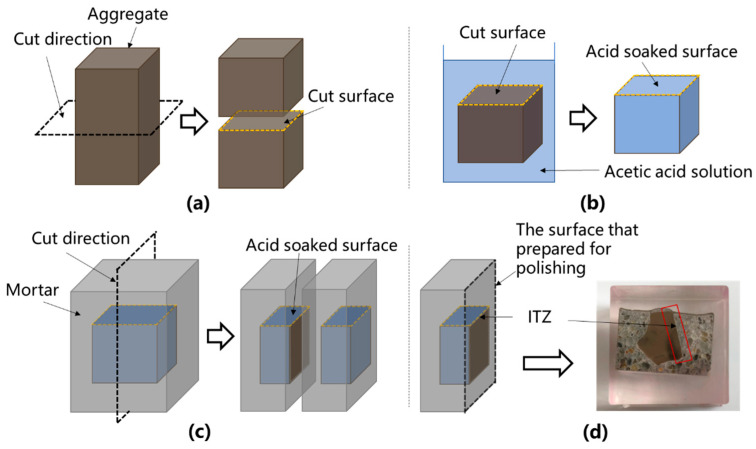
Schematic diagrams of sample preparation for the nanoindentation of ITZ: (**a**) cut natural aggregate; (**b**) immerse the cut natural aggregate in dilute acetic acid solution; (**c**) coat aggregate in mortar and then cut it; (**d**) polish cut samples for nanoindentation test.

The irregular natural aggregates were cut with an automatic section saw cutting machine (KEJING, SYJ-200, Shenyang, China) to guarantee that the aggregate’s surface had a complete cross-section, allowing for comparisons between samples, as shown in Figure 2a. The cut aggregate was reserved for the following test types. All sample preparation and testing were performed in a climate-controlled laboratory maintained at 25 ± 1 °C and 75 ± 5% relative humidity.

#### 2.2.1. Profilometer Testing Samples

The cut aggregate was vacuum-impregnated with epoxy resin and polished to provide a flat and smooth surface for testing [28,29]. Five sets of polished samples were obtained, one of which was the control group. The other four groups were submerged in 0.1, 0.2, 0.3, and 0.4 M acetic acid solution for 24 h. The samples were then ultrasonically cleaned with an alcohol solution for 2 min before being put in a vacuum drying oven at 50 °C for 3 d.

#### 2.2.2. SEM Samples

The cut aggregate was submerged in acetic acid solutions with varying concentrations (0.1, 0.2, 0.3, and 0.4 M) for 24 h, as shown in Figure 2b. The aggregates were then cleaned with water and baked in a vacuum oven at 50 °C for 3 d. A portion of the dried samples were utilized as SEM samples, while the remainder were used for nanoindentation testing. To guarantee the conductivity of the samples, the cut surfaces of the dry aggregates were sprayed with gold for 180 s before SEM observation.

#### 2.2.3. Nanoindentation Samples

The above-mentioned dry aggregate was placed in 20 mm × 20 mm × 20 mm silicone molds and then filled with fresh cement mortar, which was designed with a mix proportion of water:cement:sand = 0.55:1:2 (mass ratio). Following pouring, the silicone molds were put on a microvibration table to mechanically remove surplus air bubbles from the slurry. The samples were sealed with plastic film, left at room temperature for 24 h, demolded, and cured in a standard curing room (20 ± 2 °C, 95 ± 2% relative humidity) for 28 d. The samples were cut perpendicular to the aggregate cutting surface (Figure 2c). The cut samples were vacuum-impregnated with epoxy and subsequently well-polished to ensure a level and smooth surface for the nanoindentation test (Figure 2d) [28,29].

#### 2.2.4. Concrete Specimen

Each acetic acid solution (0.1, 0.2, 0.3, and 0.4 M) was mixed with coarse aggregate in a 2:1 volume ratio for 24 h. The aggregates were then washed with water and dried in an oven set at 50 °C until they reached a consistent weight to eliminate residual acid effects. The concrete mix design utilized OPC as the sole binder, with no supplementary cementitious materials and no plasticizers. The mix proportions by mass were cement:water:fine aggregate (natural river sand):coarse aggregate = 1.0:0.55:2.0:4.2, resulting in a water-to-cement (w/c) ratio of 0.55. The coarse aggregate comprised solely the treated or untreated limestone NCA. Following casting, the 100 mm concrete cube specimens were sealed with plastic film, left at room temperature for 24 h, and then demolded and cured in a standard curing room for 28 d.

### 2.3. Testing Methods

#### 2.3.1. Test for Aggregate

##### Physical Properties Tests

The physical properties of the original NCA before and after the acetic acid treatment, i.e., apparent density, water absorption, and crushing index, were examined following the Chinese standard GB/T 14685-2022 [30]. All tests were performed three times and averaged.

The aggregate was removed from the water after a 24 h soaking period and placed in a hanging basket that was completely submerged in water. The mass of the basket and aggregate in water (*m_h_*_2_) was then determined. The aggregate was subsequently removed from the basket, and the mass of the basket in water (*m_h_*_3_) was obtained. The aggregate was then subjected to drying in an oven at 105 ± 5 °C until a constant weight was reached, after which the mass was weighed (m*_h_*_1_). The apparent density (*ρ*) was calculated in Equation (1), where α and *ρ_w_* are the correction factor and the density of water, respectively.

(1) $$\rho =\left(\frac{m_{h1}}{m_{h1}+m_{h3}-m_{h2}}-\alpha \right)\times \rho_{w}$$

The saturated-surface-dried aggregate was obtained by drying the water-saturated aggregate with a wet towel, which was weighed (*m_j_*_1_) and then dried in an oven at 105 ± 5 °C to a constant weight *m_j_*_2_. The water absorption (*w*) was calculated by Equation (2).

(2) $$w=\frac{m_{j1}-m_{j2}}{m_{j2}}\times 100\%$$

Approximately 3 kg of dry aggregate was loaded into a circular mold in layers. Then, the aggregate in the mold was loaded to 200 kN by a pressure tester at a rate of 1 kN/s and kept the load for 5 s. Subsequently, the aggregate in the mold was poured out and weighed as *m_g_*_1_. The crushed aggregate was then sieved through a sieve with an aperture of 2.36 mm, and the remaining aggregate was also weighed as *m_g_*_2_. The crushing index (*Q_g_*) can be calculated as Equation (3).

(3) $$Q_{g}=\frac{m_{g1}-m_{g2}}{m_{g1}}\times 100\%$$

##### Profilometer Testing

Stylus profilometer: the step height between the epoxy resin and the acid-dissolved aggregate in the samples was measured using a stylus profilometer (Dektak 150, VEECO, Plainview, NY, USA) to determine the maximum corrosion depth of the acid-immersed aggregate in a specific localized region. During the test, the probe was transported in a straight line from the epoxy resin to the aggregate surface. The probe pressure was adjusted to 3 mg, and the sample was scanned for 5000 μm. The profilometer’s resolution was 0.167 μm per sample.

The 3D optical profilometer: a 3D optical profilometer (ContourGT-K 3D, Bruker, Ettlingen, Germany) was used to examine the morphology of the aggregate surface before and after acid treatment. After being placed on the sample stage, the samples were manually centered beneath the objective lens in the X and Y directions and then automatically focused in the Z direction using computer control. The samples were scanned at a rate of 28.1 µm/s with a scanning size of 200 µm × 300 µm. After obtaining the 3D surface profiles of the samples, each image was digitally analyzed, and the surface roughness of the scanned area was described using the surface arithmetic mean height (*S_a_*) according to the international standard ISO 25178 [31] to quantitatively characterize the acid dissolution of the aggregate surface in the specific region.

##### SEM Observation

A high-resolution field emission SEM (Sirion, FEI, Eindhoven, The Netherlands) was used to observe the surface morphology of the NCA before and after acetic acid treatment, operating at an acceleration voltage of 20 kV and a working distance of 5.7–6.7 mm.

#### 2.3.2. Test for Concrete

##### Workability and Compressive Strength Test

To assess the effects of different concentrations of dilute acetic acid treatment of aggregate on the workability and strength of concrete, slump and compressive strength tests were examined as per Chinese standards GB/T 50080-2016 [32] and GB/T 50081-2019 [33], respectively. The 28-day age concrete specimens were subjected to testing via a hydraulic compression tester, which was servo-controlled with a loading rate of 0.6 MPa/s. All tests were conducted in triplicate and the mean value was calculated.

##### Nanoindentation Test

**Figure 3:**
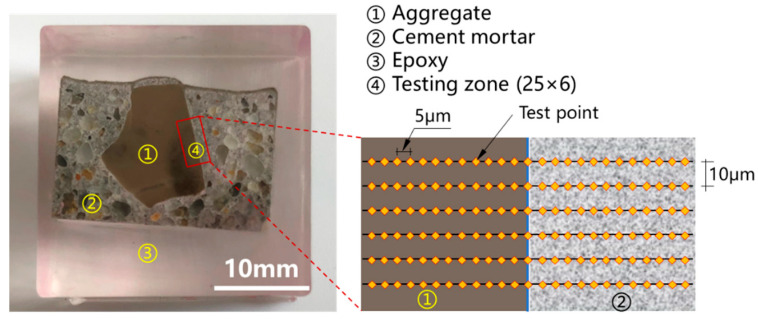
Test protocol of indentation around the interface between aggregate and mortar.

The experiment in this study was performed using a nanoindentation device equipped with a Berkovich indenter with a loading range of 0–500 mN. The nanoindentation test was loaded at a constant rate of 200 μN/s, with a maximum load of 2 mN. After that, the load was kept for 5 s and then unloaded at a constant rate of 200 μN/s. A 25 × 6 dot matrix was used to test each sample group, with 5 μm and 10 μm spacing between indentation sites on the X and Y axes. The nanoindentation area encompassed both the aggregate and the cement matrix, as seen in Figure 3. Sand should be avoided as much as possible while measuring points on a cement matrix. Furthermore, to eliminate the impact of the test sample’s roughness on the test, the sample preparation technique is followed by the backscattered electron (BSE) sample preparation procedure [34], such that the average depth of the indentation (350 nm) is more than twice as large as the roughness of the test sample [35].

## 3. Results and Discussion

### 3.1. Evaluation of the Effects of Acid Immersion on Aggregate

#### 3.1.1. Physical Properties

Table 1 shows how acetic acid immersion affects the physical characteristics of original NCAs. When the acetic acid concentration is 0.1–0.3 M, the original aggregate’s apparent density, crushing index, and water absorption remain unaltered. This suggests that a low concentration of acetic acid solution has little influence on the original aggregate. However, when the acetic acid content is 0.4 M, the apparent density of the aggregate reduces by 0.8%, but the crushing index and water absorption rise by 2.8% and 18.5%, respectively. The higher the concentration of acetic acid solution, the greater the influence on the aggregate’s water absorption. Limestone NCA is mostly composed of carbonate ores, and acetic acid interacts chemically with CaCO_3_ in the aggregate (Equation (4)), causing the aggregate surface to disintegrate and become very porous. When the concentration of acetic acid is low, it has a restricted ability to dissolve the aggregate surface. Higher doses of acetic acid dissolve and remove more mortar from the surface of recycled aggregate [21,36]. However, a high acetic acid concentration will harm the main aggregate in the recycled aggregate, reducing its physical qualities.

(4) $$CaCO_{3}+CH_{3}COOH_{\left(l\right)}\to Ca^{2+}+CH_{3}COOH_{\left(aq\right)}^{-}+CO_{2(g)}$$

#### 3.1.2. Dissolution Depth of Aggregate

**Figure 4:**
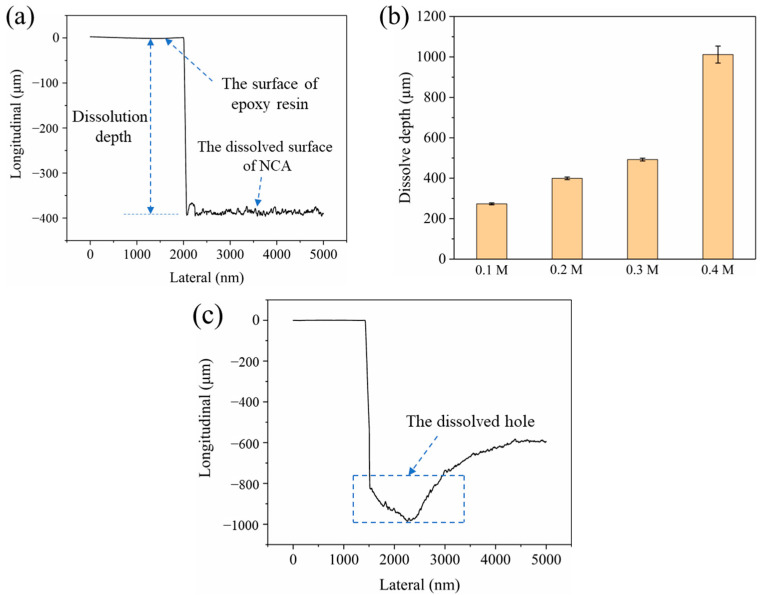
(**a**) Typical dissolution depth curve of NCA; (**b**) the dissolution depth of NCA at various concentrations of dilute acetic acid; (**c**) typical dissolution depth curve of NCA at 0.4 M acetic acid concentration.

The cured epoxy resin’s corrosion resistance prevents corrosion or deterioration when exposed to low acid concentrations (<1 M) for short periods [37]. When aggregate comes into contact with acid, a dissolution reaction occurs, resulting in a step height between the aggregate and the epoxy resin before and after the acid contact. The dissolving depth of various concentrations of acetic acid solutions on the surface of the original aggregates was measured using a stylus profiler, and Figure 4a depicts a typical dissolution curve for the original aggregate. The longitudinal probing distance of the probe on the aggregate surface after acid contact varies jaggedly within a limited range of a particular value, indicating that the acid’s corrosive activity causes uneven dissolving pits on the aggregate surface. Figure 4b shows that the average dissolution depths of original aggregates in 0.1, 0.2, and 0.3 M acid solutions were 273, 399, and 492 μm. The dissolution depths increased slowly with increasing dilute acetic acid concentration, indicating that more acid in the solution interacted with the minerals in the aggregates. However, at an acetic acid concentration of 0.4 M, the depth of dissolution increased dramatically to 1012 μm, which was attributed to the deeper dissolution craters produced in this primary aggregate. This caused substantial damage to the original aggregate, explaining the reduction of the physical properties by the excessively high acid concentration (Figure 4c).

#### 3.1.3. Surface Roughness of Aggregate

**Figure 5:**
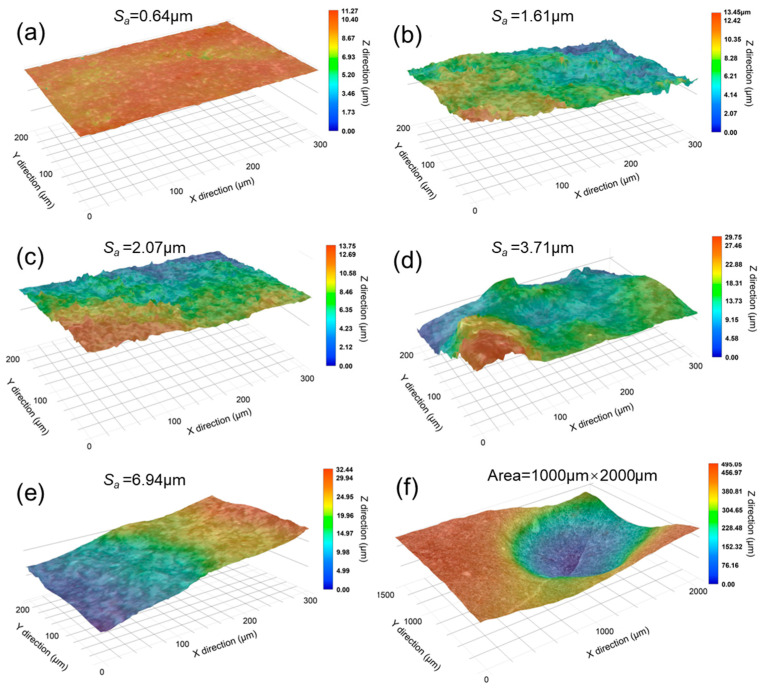
Surface optical profile morphology of NCAs immersed in various concentrations of dilute acetic acid solutions: (**a**) control aggregate; (**b**) 0.1 M; (**c**) 0.2 M; (**d**) 0.3 M; (**e**) 0.4 M, and (**f**) the magnification of aggregate surface at 0.4 M (Note: *S_a_* denotes the surface roughness of the scanned area).

The optical profilometer test was used to obtain a three-dimensional profile of the aggregate surface before and after acid immersion, as well as to calculate the surface roughness of each aggregate in the test area, which quantitatively characterizes the aggregate’s entire surface after acid erosion in comparison to the stylus profilometer. Figure 5a depicts the contour profile of the control aggregate without acid immersion, which has a smooth surface with little evident convexity. However, following soaking in various concentrations of dilute acetic acid, the aggregate surface became significantly roughened as a result of the acid’s dissolving response. In 0.1, 0.2, and 0.3 M dilute acetic acid solutions, aggregate surface roughness increased by 0.97, 1.43, and 3.07 μm (Figure 5b–d), respectively, compared to before acid immersion (Figure 5a). The original aggregate’s surface roughness increased as the acetic acid concentration rose. In comparison to the control aggregate (Figure 5a), the aggregate’s surface roughness increased sharply by 6.30 μm at 0.4 M acetic acid concentration, more than twice as much as at a 0.3 M acetic acid concentration. The increase was attributable to the formation of dissolving pits on the aggregate surface at this concentration, which is consistent with the stylus profilometer test described above and seen in Figure 5f.

#### 3.1.4. SEM Observation

**Figure 6:**
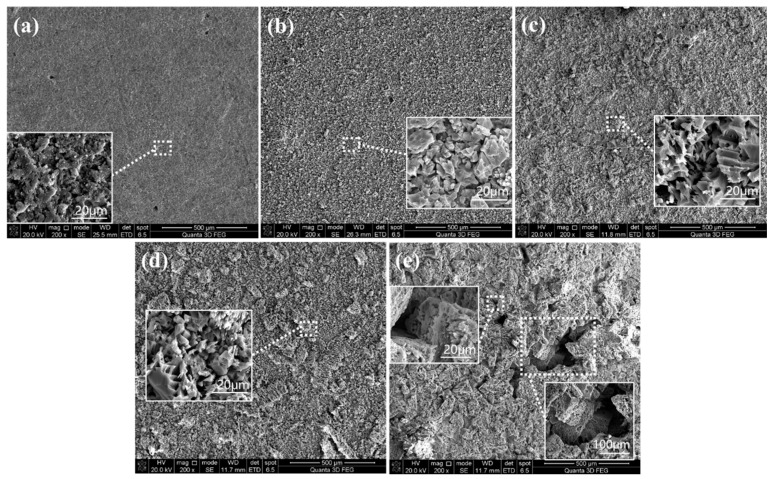
SEM observation of (**a**) untreated aggregate; (**b**), (**c**), (**d**), and (**e**) are 0.1, 0.2, 0.3, and 0.4 M acetic-acid-immersed aggregate, respectively.

SEM images of the apparent morphology of each original aggregate are shown in Figure 6. When enlarged 200 times, the surface of the aggregate appears smooth (Figure 6a). Further magnification to 5000 times reveals that the texture on the surface of the aggregate is interlocked and dense. After 200 times magnification, the surface roughness of the aggregate immersed in 0.1–0.3 M acetic acid appears to steadily increase compared to the untreated aggregate. Fine holes are shown in the aggregate’s texture after being magnified 5000 times, suggesting the presence of acid dissolution. This confirms the results of the profilometer test. However, acid corrosion did not alter the distribution of aggregate texture, and no cracks occurred on the surface of any samples. In contrast, the surface of the original aggregate treated with 0.4 M acetic acid showed significant dissolving pits and textural misalignment. This suggests that the acid solution at this concentration has a detrimental impact on the surface of the original aggregate, which is consistent with the findings of all previous research.

### 3.2. Evaluation of the Effects of Acid Immersion on Concrete

#### 3.2.1. Workability and Compressive Strength of Concrete

**Figure 7:**
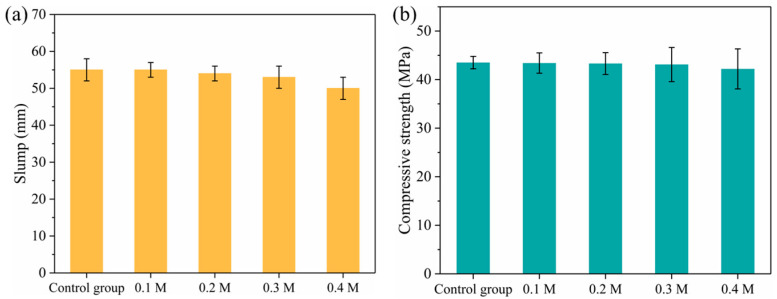
Effect of the aggregate treatment on the (**a**) workability and (**b**) compressive strength of concrete.

Figure 7 shows the impact of aggregates treated with various acetic acid concentrations on concrete workability and compressive strength. As demonstrated in Figure 7a, the slump of concrete decreased somewhat when the acetic acid content rose from 0.1 M to 0.3 M. With the 0.4 M acid solution, the slump reduced by 9.1% compared to the control group with untreated aggregate. As the concentration of acetic acid increased, the aggregate surface became rougher, increasing the aggregate’s water absorption. The rougher the aggregate surface, the greater the internal friction between the aggregates, necessitating more cement paste wrapping [38]. With the same amount of cementitious material, aggregates with rough surfaces will have lower workability than aggregates with smooth surfaces [38]. As seen in Figure 7b, the compressive strength of concrete remained relatively consistent across the range of acetic acid concentrations from 0.1 to 0.3 M when compared to the control group. When the concentration of acetic acid was raised to 0.4 M, the compressive strength of concrete fell by 3% compared to the control group. This shows that modest acetic acid concentrations have minimal dissolving effects on aggregates and do not impair concrete strength. When the concentration of acetic acid exceeds a particular threshold, its destructive action on the aggregate causes a deterioration in the mechanical qualities of concrete.

#### 3.2.2. Microproperties of ITZ

**Figure 8:**
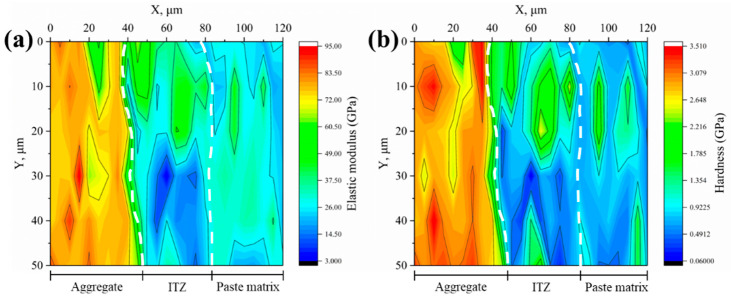
Contour maps of (**a**) elastic modulus and (**b**) hardness for the control group.

**Figure 9:**
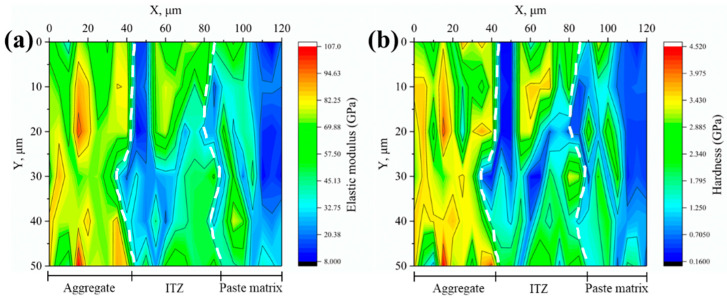
Contour maps of (**a**) elastic modulus and (**b**) hardness for the 0.1 M group.

**Figure 10:**
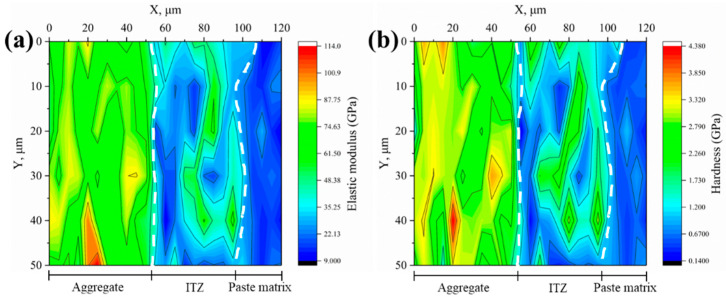
Contour maps of (**a**) elastic modulus and (**b**) hardness for the 0.2 M group.

**Figure 11:**
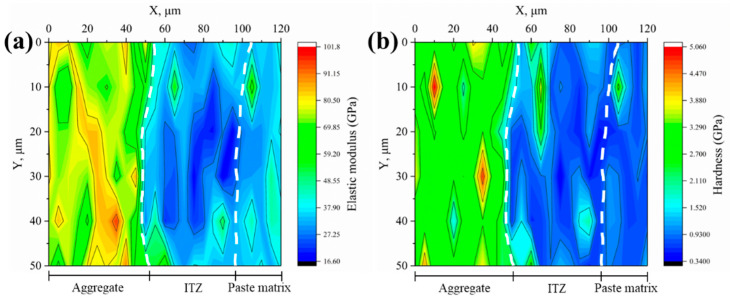
Contour maps of (**a**) elastic modulus and (**b**) hardness for the 0.3 M group.

**Figure 12:**
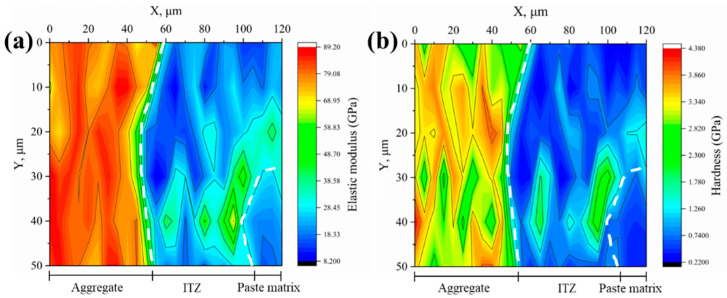
Contour maps of (**a**) elastic modulus and (**b**) hardness for the 0.4 M group.

The elastic modulus and hardness of the interfaces between various aggregates and new mortar were measured using the nanoindentation test and shown as 2D contour plots, as illustrated in Figure 8, Figure 9, Figure 10, Figure 11 and Figure 12. Aggregate has a greater modulus of elasticity and hardness, followed by ITZ and slurry. Brighter parts on the contour plots indicate higher elastic modulus or hardness, whereas darker areas indicate lower elastic modulus or hardness. As a consequence, the exact position of the interface between the aggregate and the new cement paste may be calculated, as well as the thickness of the ITZ, using the gradient change and distribution of elastic modulus or hardness in the contour plot [34,35,39]. In Figure 8, Figure 9, Figure 10, Figure 11 and Figure 12, the left dashed line represents the ITZ’s initial border, while the right dashed line represents its distal boundary. The elastic modulus contour plots and hardness contour plots show comparable color region distributions, with the ITZ distribution being particularly consistent.

**Figure 13:**
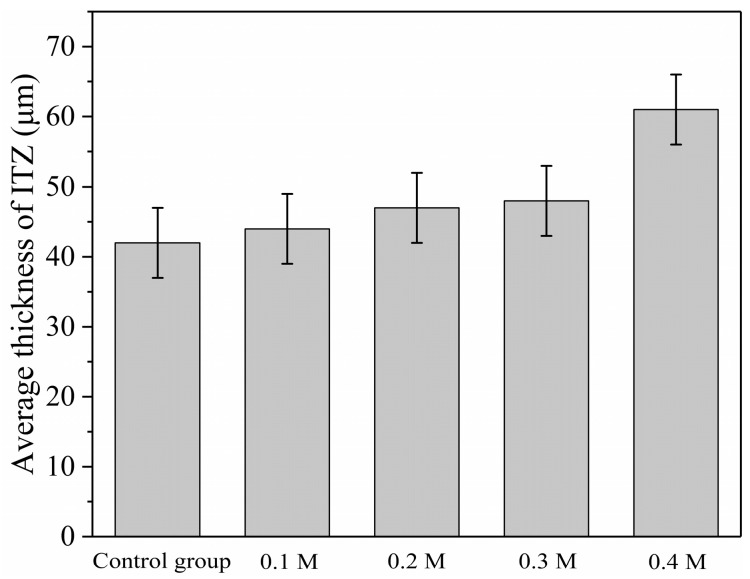
The average thickness of ITZ for different groups of samples.

The average thickness of ITZs between various aggregates and fresh mortar was calculated using the distribution of ITZs in Figure 8, Figure 9, Figure 10, Figure 11 and Figure 12, as shown in Figure 13. The average thickness of ITZ in aggregates treated with 0.1, 0.2, and 0.3 M acetic acid immersion increased by 4.76%, 11.90%, and 14.29%, respectively, as compared to aggregates not treated (control). In contrast, the aggregate treated with 0.4 M dilute acetic acid exhibited a significant increase of 45.24% in the average thickness of ITZ when compared to the control group. As a result, the acid-immersed aggregate will somewhat increase the thickness of ITZ, but only to a limited extent. When the acid content is too high, the thickness of the ITZ grows dramatically.

**Figure 14:**
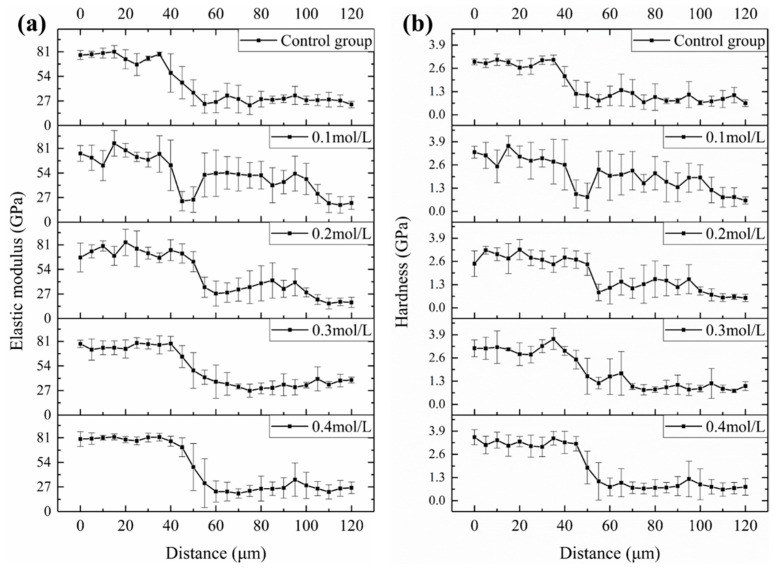
The variation curve of (**a**) average elastic modulus and (**b**) average hardness for different groups of samples along the X direction.

**Figure 15:**
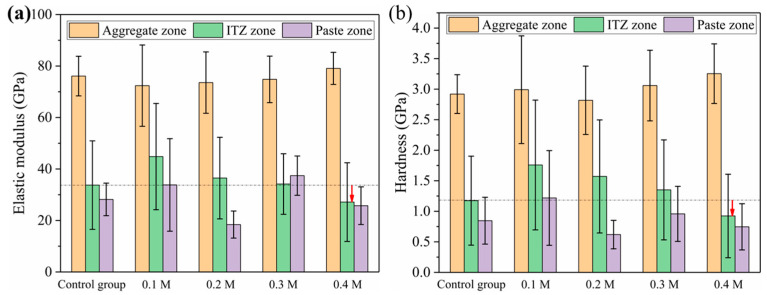
(**a**) The average elastic modulus and (**b**) average hardness of different samples in the aggregate zone, ITZ, and paste zone (the red arrow denotes the drop of elastic modulus or hardness in ITZ).

Based on the contour plots shown above, the average values of the six indentation locations in the Y direction were averaged, and the average change curves of elastic modulus and hardness in the X direction for various aggregates were generated, as illustrated in Figure 14. Aggregate has the highest elastic modulus (or hardness), with values more than 60 GPa (or >2.5 GPa), followed by ITZ and slurry, with elastic modulus (or hardness) in the range of 20–40 GPa (0.8–1.8 GPa) and 10–30 GPa (or >0.5–1.2 GPa), respectively. Figure 15 depicts the average elastic modulus and hardness values of several samples over three regions: aggregate, ITZ, and slurry. The average elastic modulus and hardness values in the ITZ area of aggregates treated with 0.1, 0.2, and 0.3 M acetic acid immersion were essentially flat or slightly enhanced as compared to aggregates not treated (control group). However, average elastic modulus and hardness values in the ITZ area of aggregates treated with 0.4 M acetic acid dropped by 18.4% and 15.9%, respectively, compared to the control group. While acid concentrations below 0.4 M leave ITZ properties unaffected, the 0.4 M treatment significantly damages the aggregate surface, increasing porosity and water absorption. This elevated surface porosity compromises C-S-H nucleation sites and traps free water needed for hydration near the interface, hindering microstructural densification within the ITZ [34,40]. Consequently, reduced hydration and pore filling lower ITZ strength and density [34,40], resulting in decreased elasticity and hardness.

However, it has been demonstrated that increasing the roughness of the aggregate surface, which enhances mechanical interlocking between the aggregate and the new mortar, can improve the microstructure of the ITZ to some extent [41]. In contrast, in this investigation, acid treatment enhanced the surface roughness of the aggregate, but this was due to acid erosion of the aggregate surface, which resulted in dissolution pits. Based on the physical properties of the original aggregates mentioned above, the results show that this aggregate with surface dissolution pits absorbs more water than the untreated aggregate, indicating that the dissolution pits increase the pore space on the aggregate’s surface. As a result, the eroded aggregate is unable to interact with the fresh mortar to provide a stronger mechanical interlocking effect. Furthermore, when acid concentration increases, the developing pattern of ITZ thickness, modulus of elasticity, and microhardness indicates that the link between aggregate and fresh mortar weakens (Table 2). When the acid concentration exceeds a particular threshold (0.3 M in this investigation), the negative impact on the ITZ becomes more pronounced. This immediately reduced the compressive strength of concrete. Furthermore, the ITZ structure has a significant impact on the transport characteristics of hazardous ions in concrete, which may affect its durability [42,43]. Notably, despite the observed ITZ degradation at high acid concentrations (≥0.3 M), the environmental advantages of acetic acid treatment remain significant. The waste solution can be neutralized with limestone powder for safe disposal or repurposed as mixing water, ensuring minimal ecological impact [21,36]. This closed-loop approach offsets the need for harsh chemical treatments while maintaining sustainability.

## 4. Conclusions

This study systematically evaluated the concurrent effects of equivalent acetic acid concentrations on both original limestone NCA within RCA and the treatment-derived concrete. The main conclusions can be drawn as follows:

(1) Acetic acid concentrations below 0.3 M preserve the structural integrity of limestone NCA, with minimal impacts on apparent density, crushing index, and water absorption, whereas exposure to 0.4 M triggers a critical degradation threshold characterized by an 18.5% surge in water absorption through millimeter-scale surface pitting.

(2) Treatment of limestone NCA with 0.1–0.3 M acetic acid preserves concrete workability and strength, whereas exposure to 0.4 M induces measurable performance declines, specifically a 9.1% reduction in slump and a 3% loss of compressive strength.

(3) After soaking the original limestone NCA in a low-concentration acetic acid solution, the thickness of the ITZ in concrete gradually increases, but it has minimal influence on the elastic modulus and hardness of the ITZ. However, as the concentration of dilute acetic acid exceeds 0.4 M, the thickness of the ITZ rises dramatically while the elastic modulus and hardness of the ITZ fall substantially.

(4) While dilute acetic acid effectively removes adhered mortar, concentrations must be constrained below 0.3 M to ensure structural preservation of both limestone NCA and concrete matrices, with 0.4 M representing the critical degradation onset point.

The identified degradation threshold (≤0.3 M acetic acid) provides a critical operational guideline for RCA processing. Maintaining this concentration range is practically feasible in industrial settings, as dilute acetic acid solutions are readily controllable using standard dosing systems. Implementing this threshold ensures effective mortar removal while preserving the essential quality of the limestone NCA, directly optimizing the performance of concrete produced with treated RCA. This approach balances treatment efficacy with aggregate integrity at a scale relevant to recycling facilities.

## Figures and Tables

**Table 1:** Physical properties of NCA within RCA.

Physical Property	Untreated Aggregate	Aggregate Soaking in Different Acid Solutions (M)			
		0.1	0.2	0.3	0.4
Apparent density (g/cm^3^)	2.62 ± 0.12	2.62 ± 0.08	2.62 ± 0.14	2.62 ± 0.11	2.60 ± 0.13
Crushing index (%)	7.2 ± 0.9	7.2 ± 0.8	7.2 ± 0.6	7.2 ± 0.5	7.4 ± 0.8
Water absorption (%)	2.7 ± 0.3	2.7 ± 0.5	2.8 ± 0.3	2.8 ± 0.5	3.2 ± 0.6

**Table 2:** Concrete properties with various aggregate.

Property	Untreated Aggregate	Aggregate Soaking in Different Acid Solutions (M)			
		0.1	0.2	0.3	0.4
Slump (mm)	55 ± 3	55 ± 2	54 ± 2	53 ± 3	50 ± 3
Compressive strength (MPa)	43.5 ± 1.3	43.4 ± 2.1	43.3 ± 2.3	43.1 ± 3.5	42.2 ± 4.1
Average ITZ thickness (μm)	42 ± 5	44 ± 5	47 ± 5	48 ± 5	61 ± 5
Average elastic modulus in ITZ (GPa)	33.74 ± 17.20	44.83 ± 20.64	36.48 ± 15.84	34.16 ± 11.77	27.14 ± 15.29
Average hardness in ITZ (GPa)	1.17 ± 0.73	1.76 ± 1.06	1.57 ± 0.93	1.35 ± 0.82	0.92 ± 0.68

## Data Availability

The original contributions presented in this study are included in the article. Further inquiries can be directed to the corresponding author.
